# An evaluation of DNA extraction methods on historical and roadkill mammalian specimen

**DOI:** 10.1038/s41598-023-39465-z

**Published:** 2023-08-11

**Authors:** Noëlie Molbert, Hamid Reza Ghanavi, Tomas Johansson, Maria Mostadius, Maria C. Hansson

**Affiliations:** 1 Centre for Environmental and Climate Science, Lund University, Ecology Building, 223 62 Lund, Sweden; 2 Department of Biology, Functional Zoology Unit, Lund University, Ecology Building, 223 62 Lund, Sweden; 3 Department of Biology, Microbial Ecology Group, Lund University, Ecology Building, 223 62 Lund, Sweden; 4 The Biological Museum, Lund University, Arkivcentrum Syd, Porfyrvägen 20, 22478 Lund, Sweden

**Keywords:** Genetics, Molecular biology

## Abstract

Guidelines identifying appropriate DNA extraction methods for both museum and modern biological samples are scarce or non-existent for mammalian species. Yet, obtaining large-scale genetic material collections are vital for conservation and management purposes. In this study, we evaluated five protocols making use of either spin-column, organic solvents, or magnetic bead-based methods for DNA extraction on skin samples from both modern, traffic-killed (n = 10) and museum (n = 10) samples of European hedgehogs, *Ericaneus europaeus*. We showed that phenol–chloroform or silica column (NucleoSpin Tissue) protocols yielded the highest amount of DNA with satisfactory purity compared with magnetic bead-based protocols, especially for museum samples. Furthermore, extractions using the silica column protocol appeared to produce longer DNA fragments on average than the other methods tested. Our investigation demonstrates that both commercial extraction kits and phenol–chloroform protocol retrieve acceptable DNA concentrations for downstream processes, from degraded remnants of traffic-killed and museum samples of mammalian specimens. Although all the tested methods could be applied depending on the research questions and laboratory conditions, commercial extraction kits may be preferred due to their effectiveness, safety and the higher quality of the DNA extractions.

## Introduction

Due to the current worldwide collapse of wild population and biodiversity overall, unraveling how organisms adapt to environmental changes is of crucial importance for conservation and management purposes. Determining the evolutionary history of wild organisms however requires sufficient genetic material over time and space. While the field of museomics is expanding due to the potential of museum samples as a vast source of DNA^1,2^, samples from live animals—mainly blood and soft tissues—are more challenging to collect, especially for endangered species. Recently, non-lethal and non-invasive sampling strategies have become increasingly popular as they alleviate needs over trapping and sedation of living animals^3^, therefore minimizing additional stress on individuals and natural populations. Among them, roadkill represents an underutilized source of molecular data for DNA-based studies. With ~ 29 million traffic-killed mammals on European roads every year^4^ and even higher numbers for birds and amphibians^5^, road mortality is one of the main causes of vertebrate species biodiversity loss^6^. Wild mammal roadkill is indeed so frequent and relatively easy to survey that it gives access to a large number of specimens of endangered and commonly encountered species. Because of the ease of sample acquisition in large quantities and across broad geographical regions, retrieving DNA from roadkill samples appears as a promising molecular ecological approach^7,8^, especially since specimen collection are decreasing^9^.

The literature on DNA extraction methods for genetic studies is extensive but focuses primarily on preserved biological material mainly from insects, plants, and birds^10–12^. While recent works have evaluated the use of roadkill to retrieve suitable DNA quality for downstream processes^8,13,14^, application of DNA extraction methods has never been simultaneously evaluated on roadkill and mammalian museum specimens. Yet combining both modern and historical samples appear highly valuable for phylogenetic reconstruction and evolutionary inferences^15^. This absence of guidelines might be related to the methodological challenges of extracting satisfactory DNA quality and quantity from differently preserved samples. Due to postmortem DNA degradation and exposure to environmental condition—such as UV radiation, high temperature and humidity—roadkill samples are more likely to contain less usable DNA compared to fresh and well-preserved samples (e.g., from blood), and be contaminated by non-target DNA^16,17^. In case of museum specimens, many factors such as the age, fixation method and conservation state could affect greatly the DNA quality of the sample, which is usually translated into fragmented DNA^2^. However, extraction methods should be appropriate across sample types and aimed to maximize yields, minimize contamination while being inexpensive and time efficient. These methods can be particularly difficult to establish when specimens are degraded or only restricted biological materials are available, making it difficult to obtain sufficient DNA.

With so many commercial extraction kits and “home-brew” protocols available, selecting the most efficient method to extract high-quality DNA from degraded and poor-quality biological material is essential to minimize destructive sampling. This is especially important in the case of restricted sampling such as for rare, threatened, or protected species. Although many different procedures of DNA isolation follow the same basic principle, choosing a suitable method for DNA extraction is crucial to ensure the quality and quantity of the isolated DNA to carry out downstream applications as well as the successful removal of contaminants, particularly for samples that are degraded and in low concentrations. The most common DNA extraction methods spanned phenol–chloroform, spin column, magnetic bead isolation, which suffer different drawbacks based on the starting biological material and the intended use of the recovered DNA. While phenol–chloroform method is often referred as the gold-standard for DNA extraction due to higher purity and longer fragments size compared to solid phase extraction methods^18,19^, the toxic nature of phenol and chloroform represent a major limitation. On the other hand, silica-based extraction methods, either spin column or magnetic bead format, can result in the carryover of PCR inhibitors into the extracts^20^ and the loss of short DNA molecules^21^. Selecting a technique to retrieve suitable DNA likely represents a compromise between the purity, yield, time, and costs of the whole process^22^, but no comprehensive study comparing the major protocols in use has been published to date for challenging archival and degraded mammalian samples.

The aim of our study was to (*i*) compare three main types of DNA extraction methods (silica spin-column, organic solvents- and magnetic bead-based) on skin samples from both traffic-killed and mammalian museum specimens, (*ii*) determine the relative yield of the different methods, and (*iii*) compare extracted DNA quantity—with the use of 2 different optical methods, either UV–Vis measurements (NanoDrop 2000 [Thermo Fisher Scientific]) or fluorescence quantification (Quant-iT PicoGreen [Thermo Fisher Scientific])—and DNA quality with the 2100 Bioanalyzer Instrument (Agilent) for average sizing in base pairs (bp), quantification, and overall sample quality control. For these purposes, road-killed European hedgehogs were collected in Southern Sweden through a citizen science approach and museum samples were obtained from the zoological collections maintained by Lund University. All over Europe, wild populations of hedgehogs *Erinaceus europaeus* have shrunk dramatically in recent years^23,24^. The main reason involves vehicular collisions as hedgehogs are among the most frequently road killed species^25^. Encountered in both urban and rural habitats all over the continent, this widespread mammal represents an opportune species to collect large-scale genetic data.

## Results

Five DNA extraction protocols were compared and selected to encompass a range of extraction methods. Methods were compared based on DNA concentration, quality, purity, as well as protocol time and costs. The DNA samples were expected to comprise *E. europaeus* DNA, as well as probable prokaryotic and eukaryotic DNA contamination from bacteria, fungi or maggots (depending on the advanced stage of decomposition) prior to hedgehog collection.

### DNA concentration

**Figure 1:**
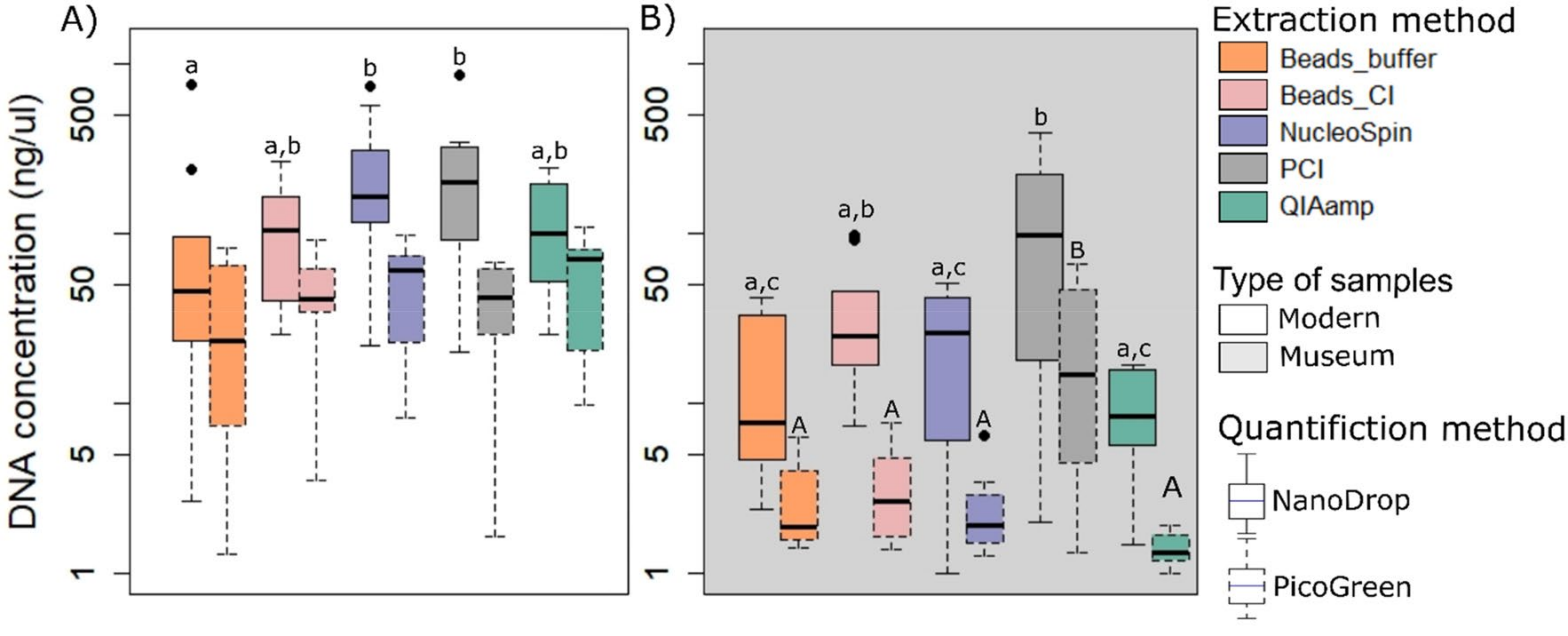
Comparison of DNA concentration (ng µL^−1^) from 5 extraction methods on (**A**) roadkill (modern) and (**B**) historical (museum) samples of European hedgehogs. Boxes extend from the 25th to the 75th percentile of each group’s distribution of values; Whiskers above and below the box indicate the 10^th^ and 90^th^ percentiles; Black horizontal line represents median values and black dot represents outliers. Capital letters indicate significant differences in DNA concentrations, evaluated with NanoDrop, among extractions methods and lower-case letters for PicoGreen measurements.

The five different DNA extraction protocols generated variable concentrations of DNA from *E.europaeus* specimens. Whatever method of quantification, median DNA concentrations vary between 23.5 ng µL^−1^ and 167 ng µL^−1^ for modern samples, and between 1.34 ng µL^−1^ and 98.9 ng µL^−1^ for museum samples. The fluorometer and NanoDrop 2000 spectrophotometer produced different readings of DNA concentration, with consistently lower DNA concentrations with PicoGreen compared to the NanoDrop 2000. On average, DNA concentrations assessed by NanoDrop 2000 was 3 times higher than concentrations measured with the fluorometer for fresh roadkill, 4 times higher for less preserved roadkill samples (*i.e.*, only skin and spines left) and over 8 times higher for museum specimens. Based on the NanoDrop 2000 spectrophotometer quantification, extraction methods produced significant differences in DNA concentrations for both modern (Linear mixed model [LMM]: *F*_(4,34.8)_ = 3.70, *p* = 0.013) and museum samples (LMM: *F*_(4,35.1)_ = 7.73, *p* < 0.001), with the PCI protocol producing the highest concentrations (Median, 202 ng µL^−1^ and 98.9 ng µL^−1^ for both modern and museum samples, respectively; Fig. 1A). While DNA concentrations of modern samples did not differ among extraction methods with the fluorometer (LMM: *F*_(4,35.05)_ = 2.17, *p* = 0.09), it varied for museum samples (LMM: *F*_(4,35.51)_ = 11.3, *p* < 0.001) with the PCI protocol yielding the highest median concentrations (Fig. 1B; 14.8 ng µL^-1^).

### DNA purity and quality

**Figure 2:**
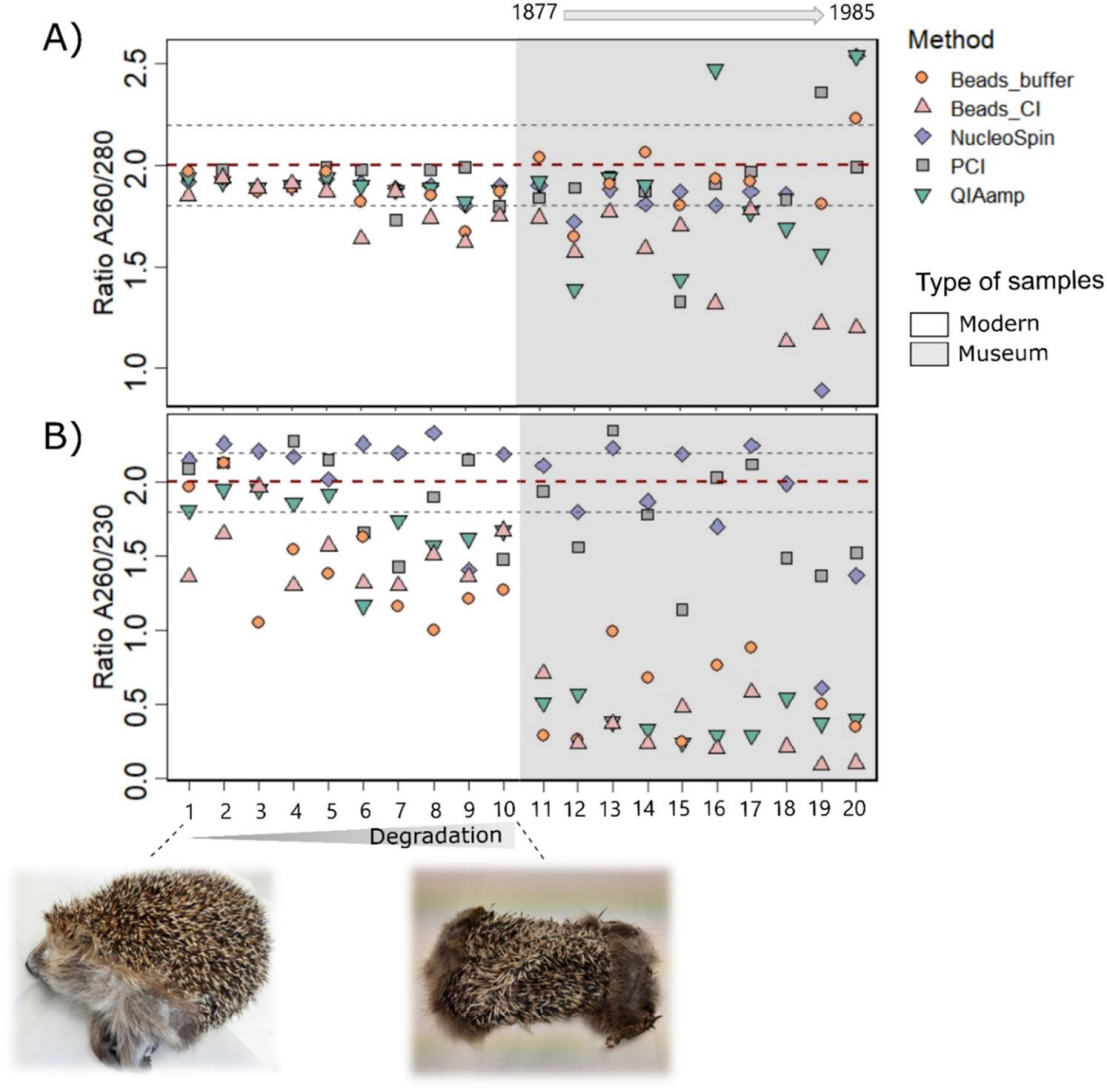
DNA quality measured by absorbance ratio (**A**) A_260/280_ and (**B**) A_260/230_ from modern (from individual #1 to 10) and museum (individual #11 to 20) samples of European hedgehogs based on five extraction methods. The dotted lines indicate the satisfactory (red) and acceptable (grey) absorbency ratios. For modern samples: from left to right, fresh to flat and dry carcasses. For museum samples: from left to right, older to more recent specimens.

DNA purity was measured using 260 nm/280 nm ratio (A_260/280_) and 260 nm/230 nm ratio (A_260/230_) NanoDrop 2000 spectrophotometer absorbance measurements. Out of the five DNA extraction methods tested on skin sample of hedgehogs, only phenol–chloroform and NucleoSpin Tissue showed satisfactory ratios, with values in the range of 1.8 to 2.0, indicating high quality DNA irrespective of the sample preservation (Fig. 2A,B).

**Figure 3:**
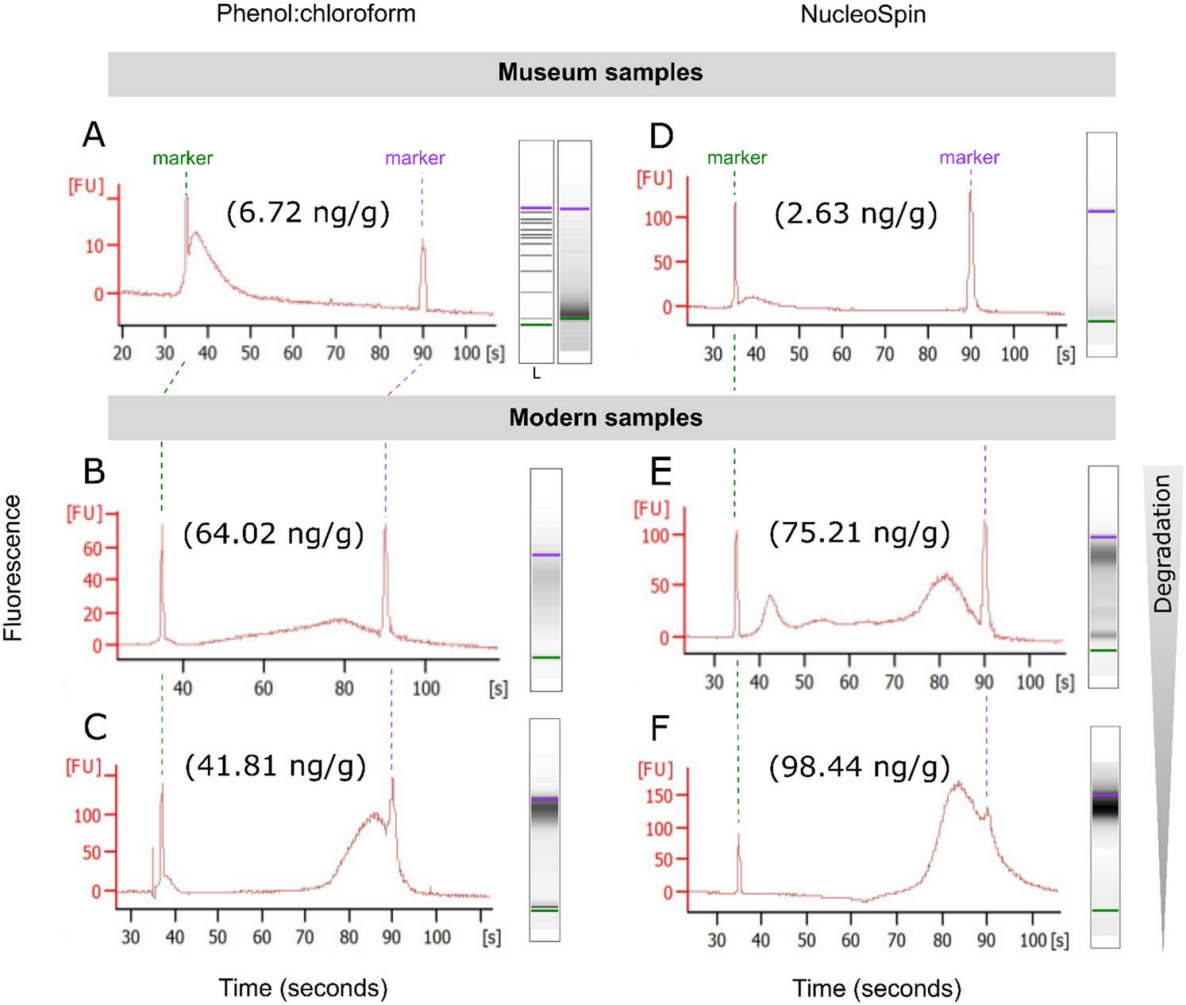
Electropherograms and gel images created by the Agilent Bioanalyzer after analyses using the Agilent DNA 12,000 kit. Shown are traces and images of DNA from museum (**A**,**D**) and roadkill (**B**,**C**,**E**,**F**) samples of European hedgehog. Samples were extracted by Phenol:chloroform (**A**–**C**) and NucleoSpin (**D**–**F**). Indicated peaks (dotted lines) represent the lower (50 bp) and the upper (17,000 bp) markers; L stands for the ladder from the kit used. DNA concentrations from PicoGreen reading are shown within parentheses for each sample.

In general, extracted DNA showed some degrees of degradation across all protocols and sample types. The distribution of the fragment lengths also seems to differ according to the sample types, with longer fragments in skin samples from freshly road-killed animals (Fig. 3C,F; 80*–*37,600 bp), followed by dry/flat hedgehogs (Fig. 3B,E; 170*–*4,000 bp) and museum specimens (Fig. 3A,D; 50*–*100 bp). There were higher inter-individual variations in fragment length profiles within and among extraction protocols, which were not related to the DNA concentration in the samples.

## Discussion

We were able to recover DNA from skin samples of European hedgehogs from historical museum collections—dry and wet specimens—as well as modern roadkill at different degradation stages. Thus, the results of our study suggest that sample preservation and age is not a limiting factor on the success of retrieving DNA, although they surely affected both DNA quality and quantity^26,27^. Recent studies on birds, amphibians and reptiles have combined historical and modern DNA^28–31^ but, to our knowledge, only few studies focused on differently preserved samples from non-human mammal species^15,32–35^. For those studies, different methods were used to extract the DNA based on the sample type (museum vs modern), which is time consuming and underlines the need for guidelines to identify appropriate DNA extraction methods on a range of differently preserved modern and historical samples. Our study on both roadkill and historical samples therefore provides a unique and valuable information for future genetic and genomic studies on mammals.

Although skin samples often yield lower DNA concentrations compared to other soft tissues^15,36^, skin tissues are the most abundant remain of traffic killed animals. In addition, study skins represent standard museum specimens. Among the biological materials available from museum collections and roadkill, skeletons represent a common source of DNA for most research, especially for studies of fossil material^37^. However, bones usually contain less DNA compared with skin clips^36^. Because roadkill and museum specimens are usually highly degraded, resulting in short DNA fragments and low concentrations, we selected existing methods that aim at maximizing the yield and size of the DNA molecules from the limited starting material. Our comparison of different DNA extraction methods shows that silica spin-column and organic-based methods (*i.e.*, NucleoSpin Tissue kit and phenol–chloroform) outperformed the magnetic bead-based protocols in amount and purity of the retrieved DNA, especially for museum samples. To date, studies comparing the efficiency of DNA extraction methods on samples from mammalian specimens are scarce and focus on various tissues, differently preserved and belonging to a wide range of species from museum collections^19,29,36^, which limit the comparisons of extraction methods with modern samples. Still, our results support the higher performance of phenol–chloroform over magnetic bead-based and QIAmp protocols regarding DNA yield and purity as well as fragment size. It is worth noting that DNA extraction methods should not be rated simply on DNA yields. On a variety of vertebrate samples, Staube et al.^38^ evidenced that the highest DNA concentrations were associated with the highest amount of exogenous DNA. While samples were cleaned prior digestion, we cannot assess the ratio of endogenous/contaminant DNA in this study, especially for roadkill hegehogs that are likely to contain extensive amounts of bacteria and worms (maggots) due to their decaying condition. For method comparison, it is however unlikely that different protocols will impact the amount of contamination by non-target DNA^19^. None of the methods used in this study has bias toward the origin of the genetic material and are therefore unable to differentiate between exogenous and endogenous DNA.

Interestingly, comparison of DNA yields from skin samples differs between absorbance and fluorescence approaches (*i.e.*, Nanodrop and PicoGreen). While DNA concentrations were fairly similar among skin samples irrespective of their preservation, for fluorometric quantitation, roadkill samples yielded on average ten times more DNA than historical specimens. Previous disagreements in DNA concentrations have been reported between these two approaches^39^, likely because all sources of nucleic acids, single- and double-stranded DNA are measured with UV spectroscopy. For sensitive applications—such as next-generation sequencing—and with low DNA concentration, it is therefore recommended to use a fluorescent based DNA concentration assay^40^. Still, disparities among extraction methods, in regards to DNA purity and quantity, are likely explained by their extraction and washing steps. For silica spin-column methods, DNA binds to a silica membrane and is washed for impurities, , while DNA is trapped and sequestered in an aqueous layer and then precipitated from solution for classic phenol–chloroform methods. Similar to commercial kits, magnetic bead-based extraction is a two-part process where DNA is first attached to the surface of silica particles, and then salts and other contaminants are removed. One common troubleshooting with spin-column based methods is the obstruction of lysate/binding buffer during the purification phase as the silica membrane is clogged with undigested sample. For phenol–chloroform, excess phenol and chloroform that remains in the sample can have significant impacts on the DNA quantitation and downstream applications^41^, although the use of Phase Lock Gel or grease will ease the total recovery of the aqueous phase therefore lowering chances of phenol or chloroform carry over. For solid-phase extraction—such as magnetic bead-based methods—a pervasive carryover of guanidine salts and/or ethanol results in low A_260/230_ values and could be greatly inhibitory to downstream analyses^20^.

As expected, the condition of roadkill animals also noticeably impacted the DNA quality, as shorter degraded fragments resulting from postmortem DNA degradation processes were observed in flat and dry hedgehog specimens. Independently of the extraction methods, short DNA fragments were obtained from the museum samples, which is typical for historical or archival samples^17^. This is mainly due to the age-related fragmentation of the DNA molecules. For this reason, phenol–chloroform protocols are usually preferred on historical samples as they allow for minimal loss of low molecular weight DNA, contrary to extraction kits. However, recent advances in museomics techniques enable the use of extremely low input of DNA for high-throughput sequencing^2,42^ and recent library prep protocols are characterized by a high tolerance for the purity of input DNA. Indeed, previous studies were able to successfully sequence target DNA^36^ and produce high-quality mammalian genome assemblies^8^ from DNA extracts with undetectable amounts of DNA using fluorometric quantification. These advances together with an appropriate selection of the DNA extraction method promise many future NGS studies using degraded and old, relatively easily accessible samples.

We demonstrate that phenol–chloroform and silica colunm-based extraction (NucleoSpin Tissue) of skin clips resulted in similar yields for mammalian museum specimens—that are up to 150 years old—and modern degraded samples such as roadkill. Although silica colunm protocols should be opted for simpler, less expensive and safer DNA extractions, it is worth mentioning that the phenol–chloroform method is the cheapest option among the methods tested here but is believed to be more hazardous. In addition, the complexity of the method requires more experienced users. Briefly, silica column protocols should be opted for simpler and safer DNA extractions when the price is not a limiting factor and high-quality DNA is needed.

## Methods

### Sample collections

Tissue samples of European hedgehogs were collected from carcasses (mainly road-kills; n = 10, hereinafter referred as modern samples) collected in the town of Lund in southern Sweden during summer 2021. Samples were collected after a plea was made through interviews in the local newspapers, radio and local tv stations to the public about sharing information on observed road killed hedgehogs. Historical samples (n = 10) of hedgehogs were obtained from the zoological collections at the Biological museum of Lund University (https://www.biology.lu.se/biological-museum/zoological-collections). Historical specimens dated from 1877 to 1988 and were either preserved with formalin injection into the body and then stored in 80% ethanol (n = 4) or with arsenic powder (n = 6) for wet and dry specimens, respectively. For both modern and historical samples, skin surfaces of 0.5 cm^2^ were collected. Each skin sample was then cut into five pieces of similar size from the same individuals allowing a comparison of the five selected methods. Modern samples were cleaned superficially in 5% sodium hypochlorite for 2 min followed by sequential washes in sterile MilliQ water (MQ) to minimize background contamination by exogenous DNA (*e.g.*, bacteria and worms).

### Materials and solutions

All pipette tips, tubes and solutions were UV-radiated to avoid non-target DNA contamination. All chemicals used in this study were analytical grade. For DNA extractions, the chloroform: isoamyl alcohol (24:1, *v*/*v*) was obtained from Thermo Fisher Scientific, ethanol (99.5%) from Solveco and the phenol: chloroform: isoamyl alcohol (25:24:1, *v*/*v*/*v*) from PanReac. The magnetic beads used in this study were Sera-Mag SpeedBeads (Cytiva 65152105050250).

### Tissue digestion and DNA extraction

**Figure 4:**
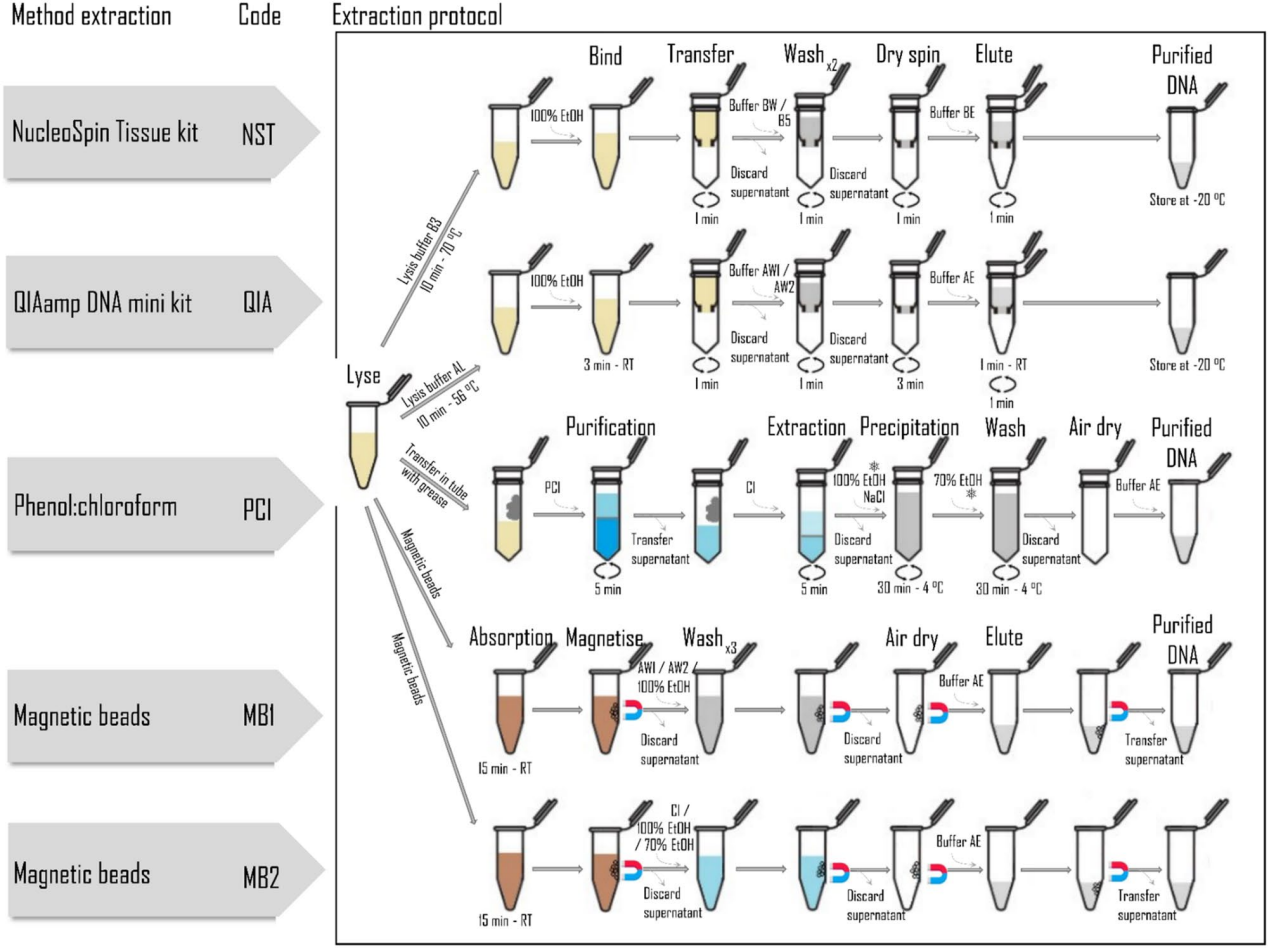
Schematic representation of the DNA extraction protocols of the European hedgehog samples.

Five DNA extraction methods, derived from an initial literature review focusing on non-human mammal samples from museum collections as well as field and roadkill surveys (Table S1), were tested for their ability to isolate and purify DNA compatible with whole-genome sequencing approaches (Fig. 4). The digestion step in our comparison was constant for all methods. In order to reduce variability in the input DNA content between different methods, a single digestion reaction was performed prior to be divided into five 200 µL aliquots. For each sample, skin tissue was cut into small pieces using a DNA-free scissor and placed in a 1.5 mL microtube. Scissors were decontaminated in bleach and washed in MQ between samples. A total of 900 µL of Buffer T1 (100 mM Tris–HCl, pH = 8, 5 mM EDTA, 0.2% SDS, 200 mM NaCl) and 100 µL of proteinase K solution (20 mg/mL, Macherey–Nagel) were added to each sample. The mixture was thoroughly vortexed and incubated overnight in a shaking incubator (56 °C, 300 rpm) until the tissue was completely lysed. The resulting 1 mL lysate was divided into 5 aliquots (200 µL each). For all methods, DNA was finally eluted in 100 µL and final volumes were stored at − 20 °C in LoBind tubes (Eppendorf) to maximize sample recovery.

#### Commercial kits

Two commercial kits using a silica-based approach, NucleoSpin Tissue (Macherey–Nagel) and QIAamp DNA Micro (QIAGEN), were tested and DNA extraction was performed according to the manufacturer’s instructions. Only the elution—performed in 2 steps—was modified to increase yield. DNA was first eluted in 50 µL of elution buffer with a 5-min incubation at room temperature. The remaining DNA was then eluted using 50 µL of buffer followed by a 15-min incubation at 70 °C.

#### Phenol–chloroform

An amount of 300 μL MQ was added to the lysate (200 μL) and mixed with the pipette prior transfer to a 2 mL sterile tube containing silicone grease (high vacuum grease 5.3 oz, Dow Corning). The grease was dispensed onto the sterile tube wall with a 60 mL syringe, approximately representing 200 μL of grease, to help stabilize the aqueous-organic interface. Then, 550 μL of phenol: chloroform: isoamyl alcohol (25:24:1, *v*/*v*/*v*) was added to the sample and shook by hand thoroughly for 5 min. A biphasic solution was formed after centrifugation at room temperature for 5 min at 10,000 rpm (~ 12,000×*g*). The upper phase was transferred to a new 2 mL sterile tube with grease. After the addition of 550 μL of chloroform: isoamyl alcohol (24:1, *v*/*v*) followed by 5 min of centrifugation at 10,000 rpm, the upper phase was transferred to a 2 mL sterile tube without grease. Subsequently, 44 μL of 5 M sodium chloride was added to the tube and DNA was precipitated in the presence of 1,100 µL cold ethanol (99.5%, − 20 °C) and stored at − 20 °C overnight. The tube was centrifuged at 10,000 rpm for 30 min at 4 °C to pellet the DNA and the solvent was discarded. This step was repeated with 1,100 µL cold ethanol (70%, − 20 °C). The supernatant was then discarded, and the pellets were air dried at room temperature for few minutes. Air dried DNA pellets were resuspended in 100 µL TE buffer, placed at 55 °C for 10 min to dissolve DNA and stored at − 20 °C until used.

The last two methods were based on magnetic DNA affinity beads (Sera-Mag SpeedBeads, Cytiva) using different cleaning approaches. The preparation of a working solution of the speedbeads follows the method described by Twort et al.^2^.

#### Magnetic beads and commercial washing buffers

A total amount of 500 μL of SpeedBeads solution was added to 200 μL lysate and incubated at room temperature for 5 min. The solution was shaken by hand and placed on a magnetic rack. The solution was allowed to rest for 3 min or until completely clear and the supernatant was discarded. Subsequently, the magnetic beads were washed three times starting with the wash buffers AW1, AW2 (QIAamp DNA Micro, QIAGEN) and ethanol (100%) as follows. First, 600 μL wash buffer AW1 were added to the tubes, mixed with a pipette off rack and placed on the magnetic rack again until clear. This step was repeated with 600 μL wash buffer AW2. Then, 800 μL of 100% ethanol were added to the tubes, incubated at room temperature, and the supernatant was discarded without disturbing the beads. With the tubes still on the rack, the beads were dried off at room temperature for 15 min with open caps before adding 100 μL AE buffer (QIAamp DNA Micro, QIAGEN) directly on the beads. Note that over drying is acceptable to evaporate all the ethanol. Tubes were taken out of the magnetic rack, thoroughly resuspended in the AE buffer and incubated for 15 min at room temperature. As a final step, the tubes were placed back on the magnetic rack until clear, the supernatant was transferred to a new 1.5 mL LoBind tube and stored at − 20 °C.

#### Magnetic beads and chloroform

A total amount of 500 μL working solution of speedbeads, which can be used for both aqueous and organic solvents^43^*,* were added to 200 μL lysate for DNA binding. The mixture was incubated on a thermoshaker (300 rpm) at room temperature (25 °C) for 15 min. The solution was then placed in a magnetic rack for 3 min or until clear, and the supernatant was discarded. Subsequently, 700 μL of chloroform: isoamyl alcohol (24:1, *v*/*v*) were added to the tube and mixed off rack with the pipette and placed back on it. The supernatant was discarded after clear solution. While on the magnetic rack, 800 μL of 100% ethanol was added to the tubes and the supernatant was discarded after 5 min of incubation at room temperature. This step was repeated with 80% ethanol. Tubes were air dried at room temperature (25 °C) with open caps paying attention to not carry over ethanol. After adding 100 μL AE buffer (QIAamp DNA Micro, QIAGEN) to the tubes—placed on the magnetic rack—the beads were thoroughly resuspended. The mixture was incubated for 15 min at room temperature prior to be placed on the rack. Finally, the supernatant was transferred to a new 1.5 mL LoBind tube and stored at − 20 °C.

### Qualitative and quantitative assessment of extracted DNA

The DNA quantities were measured using 2 different optical methods, either UV-V measurements (NanoDrop 2000 [Thermo Fisher Scientific]) or fluorescence quantification (Quant-iT PicoGreen [Thermo Fisher Scientific]). For DNA quality we used the 2100 Bioanalyzer Instrument with the Agilent DNA 12000 Analysis Kit and 1 μL of sample for average sizing in base pairs (bp), quantification, and overall sample quality control. DNA purity was measured using the ratio of different wavelengths (260 nm/280 nm: ratio A_260/280_ and 260 nm/230 nm ratio: A_260/230_) on a NanoDrop 2000 spectrophotometer. The optimal values indicating high quality DNA are 1.8 for A_260/280_ and 2.2 for A_260/230_, respectively. Ratios outside these values reflect the presence of UV-absorbing materials such as RNA, proteins and phenolic compounds. Blanks were performed using the respective elution buffer for the extraction methods (AE buffer; QIAamp DNA Micro, QIAGEN). However, UV spectrophotometers are not sensitive enough to detect small amounts of DNA. PicoGreen is another DNA quantitation method for the detection of double-stranded DNA (dsDNA). Based on a fluorescent dye, which preferentially binds to dsDNA, PicoGreen measurements provide more specific results than UV spectrophotometer. Finally, a chip-based capillary electrophoresis method (*i.e*., Agilent Bioanalyzer) was used to measure the precise size distribution and degree of DNA fragmentation.

### Statistical analysis

Data was first checked for normality and homogeneity of variances. Linear mixed model (LMM) analyses were conducted using the *lme4* and *lmerTest* packages^44,45^ in R v. 3.3.2 software^46^ to compare the quality and quantity of extracted DNA following different protocols. Log-transformed DNA concentrations (n = 98), ratio A_260/230_ (n = 98) and ratio A_260/280_ (n = 98) were used as dependent variables and method (five-level factor) as an explanatory variable. Sample ID treated as random effects to control for repeated measures of individuals. Pairwise comparisons of differences among DNA extraction methods (NucleoSpin, PCI, QIAamp, Beads_buffer and beads_CI) were tested using the *multcomp* package^47^ implemented in R. A significance level of α < 0.05 was used for all tests.

### Supplementary Information

Supplementary Table S1.

## Data Availability

The data can be shared under request to the e-mail address: noelie.molbert@uccon.edu.
