# Supplementary material for: An evaluation of DNA extraction methods on historical and roadkill mammalian specimen
Source: Sci Rep. 2023 Aug 11;13:13080. doi: 10.1038/s41598-023-39465-z (PMC10421861; doi:10.1038/s41598-023-39465-z)
Supplement: Supplementary file 1 — Supplementary Table S1. [file 41598_2023_39465_MOESM1_ESM.docx]

Table S1. Literature review on DNA extraction methods of non-human mammal samples.

| Sample type | Genus/Species | Tissue type | Extraction method | Method evaluation | Yield | Fragment size (bp) | Comment | Reference |
| --- | --- | --- | --- | --- | --- | --- | --- | --- |
| **Museum** | *Guerlinguetus brasiliensis* | Skin, muscle | Phenol-chloroform | Qubit fluorometer | nc | nc |  | 10.1186/s12862-020-01639-y |
|  | Conepatus, Gerbilliscus and Gerbillus, Ailurops, Phalanger, Strigocuscus, and Trichosurus | Skin, claw, bone,  osteaocrust | Phenol-chloroform | Qubit fluorometer, BioAnlayzer | 0.1–43 (ng/µl) | 71–91 |  | 10.1093/jmammal/gyy080 |
|  | *Peromyscus mekisturus* | Skin | DNeasy® Blood & Tissue kit,  Qiagen | Qubit fluorometer | nc | nc |  | 10.3389/fevo.2022.930356 |
|  | Mustela nivalis | Skin | Dabney et al. 2013 | nc | nc | nc |  | 10.1093/molbev/msab177 |
|  | *Ursus deningeri* | Bone | silica-based protocl | Nanodrop | nc | 35-150 | Fossil | 10.1073/pnas.1314445110 |
|  | *Panthera leo* | Tooth | Dabney et al. 2013 | Qubit fluorometer | 0.267 (ng/µl) | nc |  | 10.1186/1471-2148-14-70 |
|  | *Canis lupus signatus* | Skin, bone | Dabney et al. 2013 | Qubit fluorometer, Bioanalyzer | 84–3,812 (ng/µl) | 42–145 |  | 10.3389/fevo.2022.970249 |
|  | *Hyperacrius fertilis* | Skin, Skull | Phenol-chloroform | Qubit fluorometer,  Bioanalyzer | nc | nc |  | 10.7717/peerj.10364 |
|  | Odocoileus hemionus | Tooth, anther, bone | Phenol-chloroform | na | nc | nc |  | 10.1093/jhered/esab013 |
|  | *Panthera pardus* | Bone, tissue fragment | DNeasy® Blood & Tissue kit,  Qiagen | na | nc | nc |  | 10.1080/24701394.2017.1307973 |
|  | *Paguma larvata* | Hair | IsoHair kit | Nanodrop | 4526.6 ± 1781.5 ng | 282-881 | Roadkill and fresh samples | 10.4238/2013.November.11.1 |
|  | Panthera tigris | na | Phenol-chloroform, silica-based purification | na | nc | < 400 |  | 10.1371/journal.pbio.0020442 |
|  | Mazama genus | Bone | DNeasy® Blood & Tissue kit,  Qiagen | Nanodrop | 443^1^  (ng/μL) | nc |  | 10.1590/1678-4685-gmb-2019-0008 |
|  | *Physeter macrocephalus* | Teeth | Home-brew silica-based protocol | na | nc | 550-800 |  | 10.1046/j.1471-8278.2001.00027.x |
|  | *Pan troglodytes verus, Crocuta crocuta, Hyaena hyaena, Parahyaena brunnea* | Teeth, bone, coat, soft tissue | GuSCN/silica protocol, Tris/NaCl buffers/ isopropanol | na | nc | 214-414 |  | 10.2144/04365st05 |
|  | Rattus | Skin, skull tissue | DNeasy® Blood & Tissue kit,  Qiagen | na | nc | nc |  | 10.1093/jhered/esaa014 |
|  | Ziphiidae | Teeth, bone | Adapted silica-based method | na | nc | nc |  | 10.1093/jhered/esh054 |
|  | Smutsia, Manis, Phataginus | Muscle, skin | DNeasy® Blood & Tissue kit,  Qiagen | na | nc | nc |  | 10.1093/jhered/esx097 |
|  | Viverricula indica | na | DNeasy® Blood & Tissue kit,  Qiagen | na | nc | nc |  | 10.1093/jhered/esw085 |
|  | *Paguma larvata* | Hair | IsoHair kit, Nippon Gene | na | 995-7428 ng | nc |  | 10.4238/2013.November.11.1 |
|  | Enhydra lutris | Bone | DNeasy® Blood & Tissue kit,  Qiagen | na | nc | nc |  | 10.1371/journal.pone.0032205 |
|  | *Macaca fascicularis fascicularis* | Dry muscle | First-DNA All Tissue kit, Gen-Ial; GuSCN/silica protocol | Nanodrop, Qubit fluorometer | nc | nc |  | 10.1186/s12864-015-1437-0 |
|  | *Physeter macrocephalus* | Teeth | Adapted silica-based method | na | nc | <800 |  | 10.1046/j.1471-8278.2001.00027.x |
|  | *Arctocephalus townsendi* | Bone | DNeasy® Blood & Tissue kit,  Qiagen | na | nc | nc |  | 10.1093/jhered/esh018 |
|  | *Dipodomys panamintinus* | Skin | Phenol-chloroform | Agarose gel | nc | nc |  | 10.1007/BF02109479 |
| **Modern** | *Otocyon megalotis, Proteles cristatus* | Ear tissue, muscle, salivary glands | DNeasy® Blood & Tissue kit,  Qiagen | na | nc | nc | Roadkill samples | 10.7554/eLife.63167 |
|  | *Guerlinguetus brasiliensis* | Skin, muscle | DNeasy® Blood & Tissue kit,  Qiagen | Qubit fluorometer | nc | nc | Ethanol-preserved samples | 10.1186/s12862-020-01639-y |
|  | Cervidae | Embryo, placenta | Phenol-chloroform | Nanodrop, agarose gel | 5.45 ± 0.12 (μg/g) | 16.4 × 10^3^ |  | 10.1080/24701394.2016.1186666 |
|  | *Erinaceus europaeus* | Hair follicles, muscle | Realpure Genomic DNA extraction kit | na | nc | nc | Roadkill and fresh samples | 10.1007/s42991-022-00235-5 |
|  | *Sus scrofa* | ear, skin tissue, hair, and/or feces | DNeasy Plant Mini Kit, Qiagen | Nanodrop, Qubit 4 Fluorometer | nc | nc | Dead animals and fresh samples | 10.3390/su14127463 |
|  | *Microtus oeconomus* | Toe clips | QIAamp® DNA Stool mini kit | Nanodrop | nc | nc |  | 10.3390/genes13030434 |
|  | Panthera tigris | Blood, muscle | Phenol-chloroform, silica-based purification | na | nc | nc |  | 10.1371/journal.pbio.0020442 |
|  | Ailuropoda melanoleuca | Blood | DNeasy® Blood & Tissue kit,  Qiagen | na | nc | nc |  | 10.1093/molbev/msu210 |
|  | *Puma yagouaroundi* | Skin | Phenol-chloroform | Nanodrop, agarose gel | nc | nc | Biopsy | 10.1093/jhered/esab036 |
|  | *Equus asinus, Equus grevyi, Equus quagga boehmi* | Blood, hair | Nucleon HT Genomic DNA Extraction Kit, Phenol-chloroform | na | nc | nc |  | 10.1038/ncomms1644 |
|  | *Cervus nippon* | na | DNeasy® Blood & Tissue kit,  Qiagen | na | nc | nc | Roadkill and fresh samples | 10.3390/ani12080998 |
|  | *Pteropus* | Skin | Phenol-chloroform | na | nc | nc | Preserved in ethanol (95%) | 10.1023/A:1015161305843 |
|  | Ursus maritimus, Ursus arctos | na | DNeasy® Blood & Tissue kit,  Qiagen | Bioanalyzer | nc | nc |  | 10.1371/journal.pgen.1003345 |
|  | Rattus | Skin, liver, skull tissue | DNeasy® Blood & Tissue kit,  Qiagen | na | nc | nc |  | 10.1093/jhered/esaa014 |
|  | *Petaurus breviceps* | gonads | Puregene^®^ Tissue Kit, Qiagen | Agarose gel | nc | nc |  | 10.7717/peerj.6180 |
|  | *Gasteracantha* | na | DNeasy® Blood & Tissue kit,  Qiagen | na | nc | nc | 95% ethanol | 10.7717/peerj.8976 |
|  | *Dasypterus ega, Dasypterus intermedius* | Wings | Ammonium acetate/isopropanol precipitation | na | nc | nc | Carcasses,  95% ethanol | 10.7717/peerj.10348 |
|  | *Naemorhedus caudatus* | Blood, skeleton | Gentra Puregene Tissue Kit, Qiagen; DNeasy® Blood & Tissue kit,Qiagen | na | nc | nc |  | 10.1266/ggs.90.31 |
|  | *Lynx lynx dinniki* | Scat, ear | Innu Prep Stool DNA kit, Analytik Jena; QIAamp DNA Stool Mini kit, Qiagen | na | nc | nc | Roadkill and fresh samples, 70% ethyl alcohol | 10.1080/24701394.2018.1445240 |
|  | *Tapirus bairdii* | Blood | DNeasy® Blood & Tissue kit,  Qiagen | na | nc | nc | 95% ethanol | 10.7717/peerj.13440 |
|  | Sciurus vulgaris | Ear tissue | Nucleospin tissue kit, Macherey Nagel | na | nc | nc | Carcasses, Roadkill samples; 90% ethanol | 10.1371/journal.pone.0047607 |
|  | Smutsia, Manis, Phataginus | Muscle, tongue | Phenol-chloroform | na | nc | nc |  | 10.1093/jhered/esx097 |
|  | Viverricula indica | Hair | CTAB-based protocol | na | nc | nc | Roadkill and field survey | 10.1093/jhered/esw085 |
|  | *Ceratotherium simum, Diceros bicornis* | Blood, horn | Prepfiler kit on Kingfisher Magnetic Particle Processor | Nanodrop | 17.8-247 ng/ul | nc |  | 10.1016/j.fsigen.2013.04.003 |
|  | Manis pentadactyla pentadactyla | Scale, muscle | FM Kit, Wako Pure Chemical; Blood and Tissue Genomic Mini Kit ,Viogene | na | nc | nc |  | 10.1016/j.fsigen.2010.06.003 |
|  | *Vulpes vulpes* | Muscle | DNeasy® Blood & Tissue kit,  Qiagen | Nanodrop | nc | nc | Carcasses | 10.1371/journal.pone.0184349 |
|  | *Paguma larvata* | Hair | IsoHair kit, Nippon Gene | na | 1851-8586 ng | nc | Roadkill and capture | 10.4238/2013.November.11.1 |
|  | Enhydra lutris | Flipper plugs | Phenol-chloroform | na | nc | nc | 100% ethanol | 10.1371/journal.pone.0032205 |
|  | *Macaca fascicularis fascicularis* | Blood, fecal sample | First-DNA All Tissue kit, Gen-Ial | Nanodrop | nc | nc |  | 10.1186/s12864-015-1437-0 |
|  | *Dipodomys panamintinus* | Liver | Phenol-chloroform | na | nc | nc |  | 10.1007/BF02109479 |
